# Supplementary material for: Chromatin assembly by the histone chaperone HIRA facilitates Human Papillomavirus replication
Source: bioRxiv. 2025 Sep 5:2025.09.05.674520. Preprint. [Version 1] doi: 10.1101/2025.09.05.674520 (PMC12466780; doi:10.1101/2025.09.05.674520)
Supplement: Supplement 1 [file media-1.pdf]

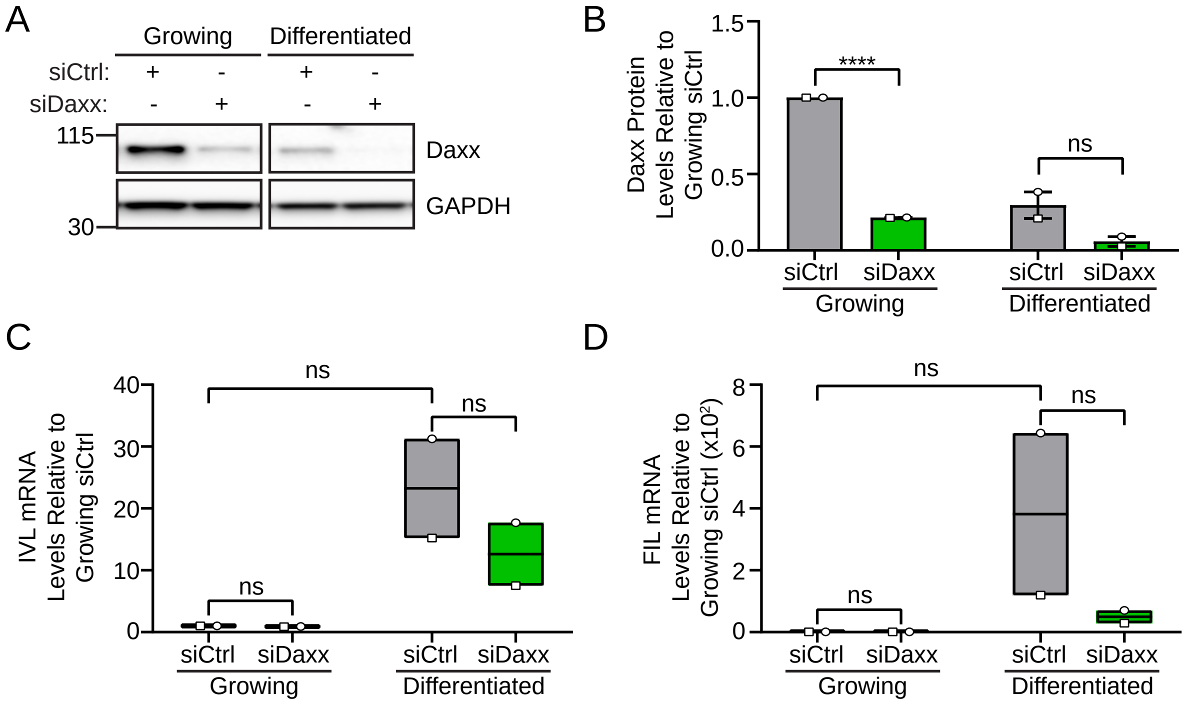

Supplementary Figure 1: Depletion of Daxx results in impaired keratinocyte differentiation in 9E cells.

Cells were plated at low density and transfected with 25 nM control (siCtrl) or Daxx (siDaxx) siRNA after 24 hours. Growing and differentiated cells were collected two and eight days post transfection, respectively. **A.** Representative immunoblots of growing and differentiated 9E siCtrl and siDaxx cells, visualizing the protein levels of Daxx and GAPDH (n=2). **B.** Quantitation of panel A, fold change in Daxx levels normalized to GAPDH relative to growing siCtrl (n=2). **C-D.** qRT-PCR using reverse transcribed RNA from growing and differentiated 9E cells transfected with siCtrl or siDaxx, showing the fold change in involucrin and filaggrin cellular transcripts normalized to PPIA within its own condition relative to growing siCtrl (n=2). The same siCtrl samples were utilized in Figure 7. For panel B, error bars represent the range. For panels C-D, floating bars represent minimum and maximum values, and the line indicates the mean. Shapes represent independent experiments. Statistical significance was calculated using an unpaired (Panel B) or paired (Panel C-D) student's t-test. Ns, not statistically significant, ****, P ≤ 0.0001.

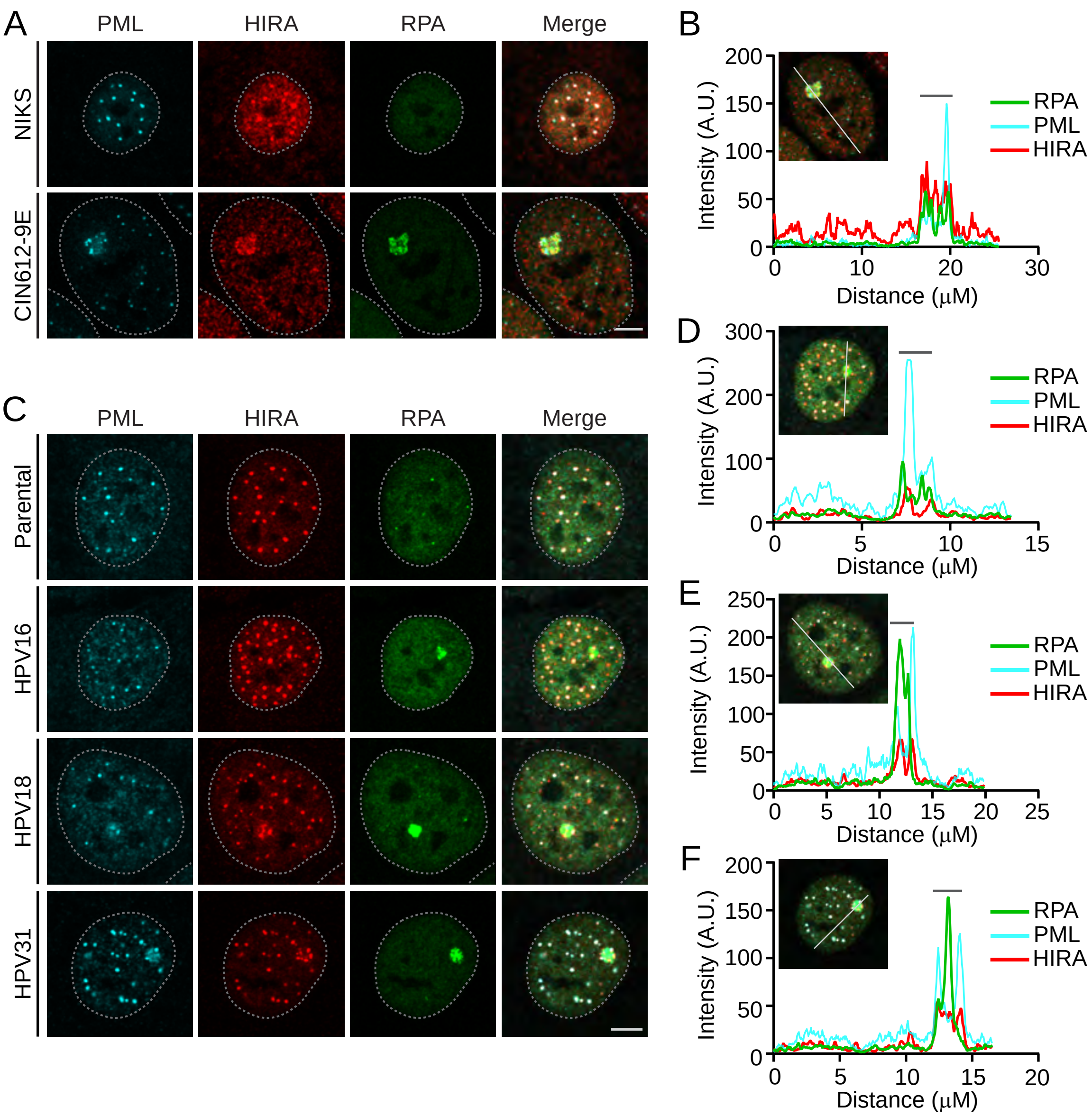

Supplementary Figure 2: HIRA localizes with PML-NBs and late replication foci in keratinocytes containing various HR-HPV genomes.

A. Representative immunofluorescence staining of PML (pseudo-colored cyan), HIRA (pseudo-colored red), and RPA (green) in differentiated NIKS and 9E cells from three independent experiments. **B.** Fluorescence intensity line scan obtained by drawing a line through the nucleus in panel A in Leica LAS X software. Gray bar above the scan indicates the replication foci. **C.** Representative immunofluorescence staining of PML (pseudo-colored cyan), HIRA (pseudo-colored red), and RPA (green) in differentiated HPV negative HFK, and HFK cells containing HPV16, HPV18, and HPV31 from two independent experiments. **D-F.** Fluorescence intensity line scan obtained by drawing a line through the nucleus in panel C for nuclei containing HPV16, HPV18, and HPV31 genomes, respectively. For all images, a gray dotted line outlines the nucleus defined by DAPI staining. Scale bar, 5 μm.

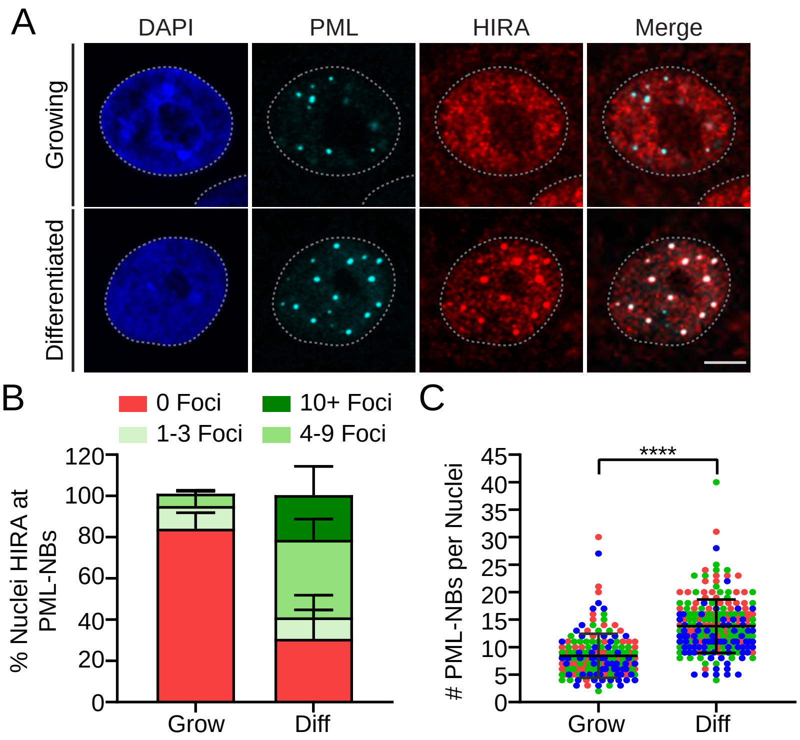

Supplementary Figure 3: HIRA localization at PML-NBs is increased with differentiation in NIKS cells.

A. Representative immunofluorescence staining of PML (pseudo-colored cyan), HIRA (pseudo-colored red), and RPA (green) in growing and differentiated NIKS cells from three independent experiments. A gray dotted line outlines the nucleus defined by DAPI staining. Scale bar, 5 μm. These images are derived from experiments also represented in Supplementary Figure 1 Panel A. **B.** The percentage of nuclei where HIRA was associated with PML-NBs, categorized by the number of PML-NBs containing HIRA (0 Foci, 1-3 Foci, 4-9 Foci, or 10+ foci). **C.** The number of PML-NBs per nuclei. For panel B and C quantitation, a minimum of 60 nuclei were manually assessed per condition from maximal projections of 3D optical slices. Error bars represent ± standard deviation of the mean and statistical significance was calculated using an unpaired Mann-Whitney test. ****, $P \leq 0.0001$.

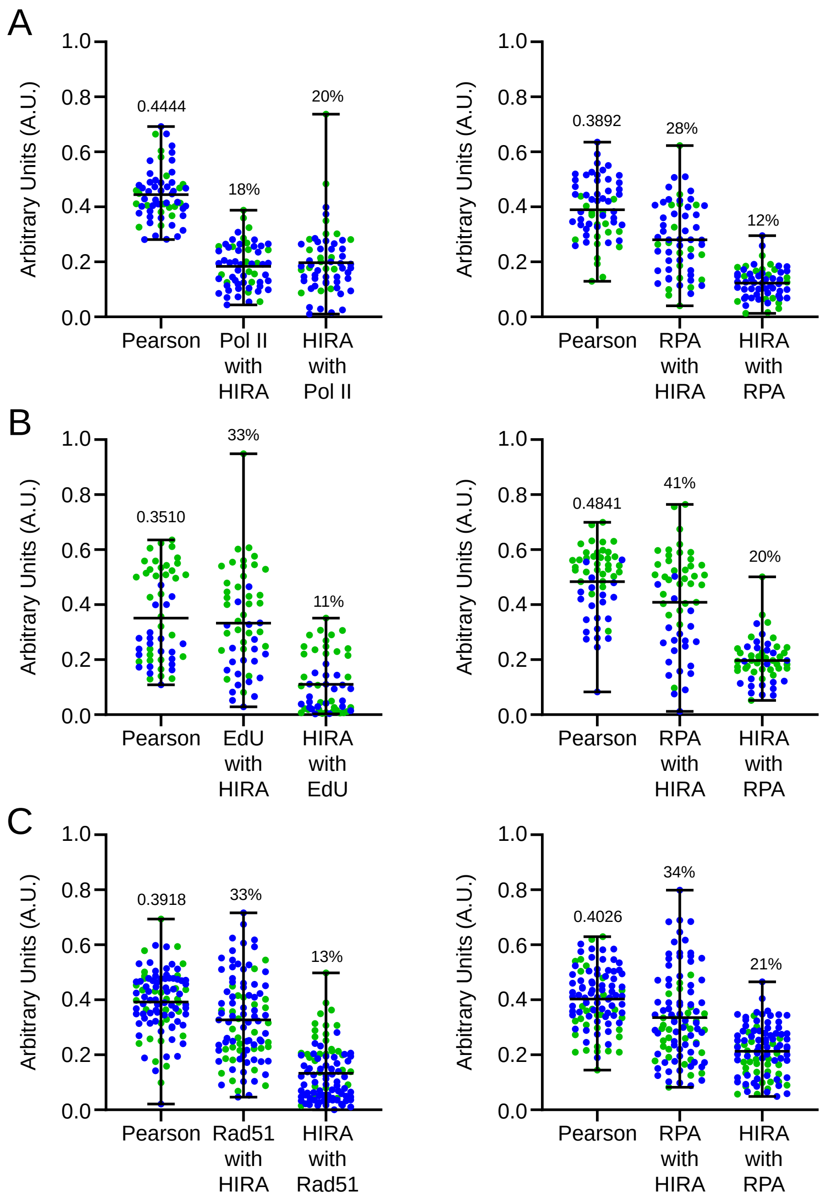

Supplementary Figure 4: HIRA does not colocalize with any singular host factor at sites of HPV31 replication.

Z-Stack images were processed in Huygens Essential (cross-talk correction and deconvolution) and colocalization analysis was performed in Imaris within a 3D region of interest (ROI), the replication foci, as defined by the RPA signal. The Pearson's Coefficient in ROI volume and the Threshold Manders' coefficients are shown from images represented in Figure 2. **A.** Colocalization analysis between RNAPIIS2 and HIRA (left) and RPA and HIRA (right) from images represented in Figure 2A-B from a minimum of 11 nuclei/18 replication foci per replicate (n=2). **B.** Colocalization analysis between EdU and HIRA (left) and RPA and HIRA (right) from images represented in Figure 2C-D from a minimum of 12 nuclei/21 replication foci per replicate (n=2). **C.** Colocalization analysis between Rad51 and HIRA (left) and RPA and HIRA (right) from images represented in Figure 2E-F from a minimum of 10 nuclei/30 replication foci per replicate (n=2). Circles denote individual replication foci and colors represent independent experiments. Shown are the mean values and error bars represent the range.

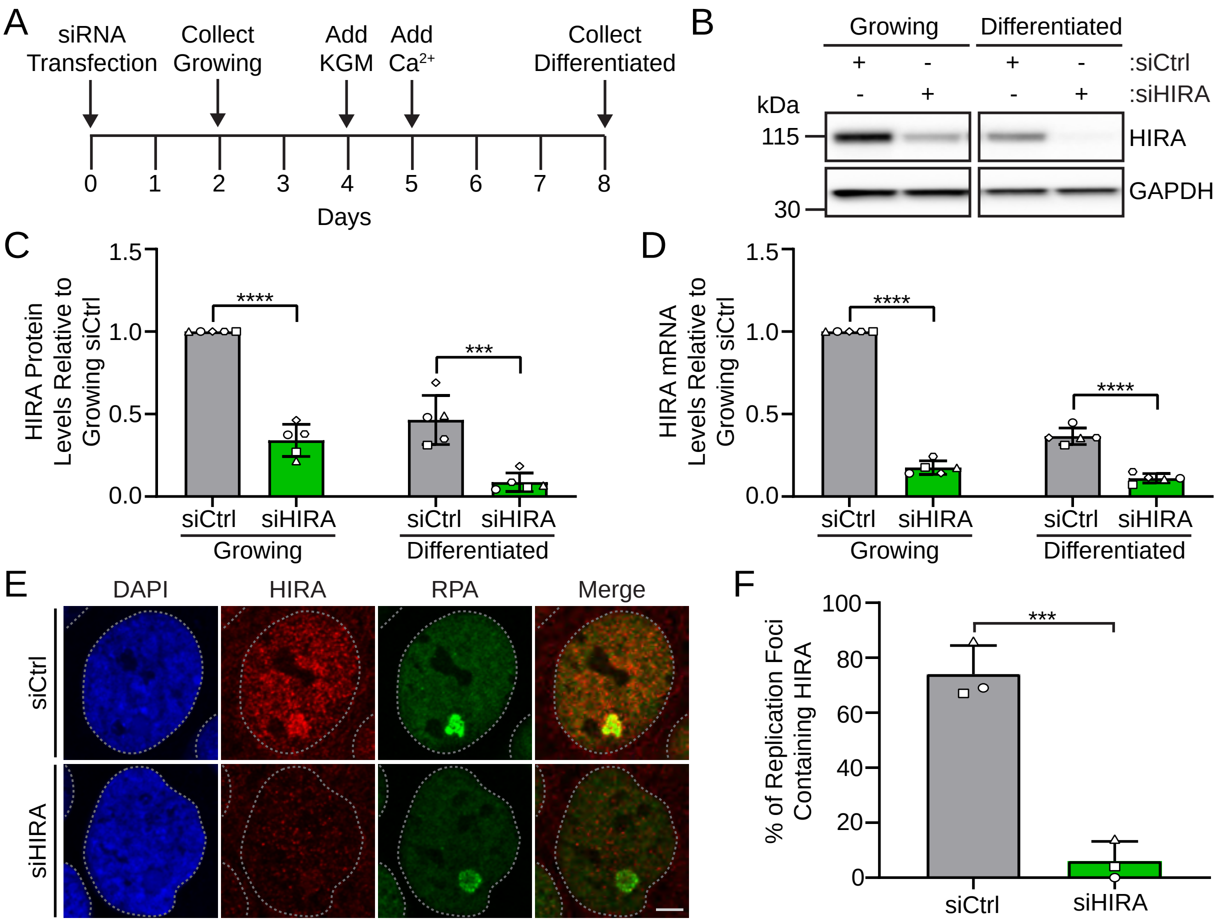

Supplementary Figure 5: HIRA is efficiently downregulated by siRNA in growing and differentiated 9E cells.

A. Timeline of siRNA knockdown in 9E cells. Cells were plated at low density and transfected with 25 nM control (siCtrl) or HIRA (siHIRA) siRNA after 24 hours. Growing and differentiated cells were collected two, and eight-days post transfection, respectively. **B.** Representative immunoblots of growing and differentiated 9E siCtrl and siHIRA cells, visualizing the protein levels of HIRA and GAPDH (n=5). **C.** Quantitation of panel B, fold change in HIRA levels normalized to GAPDH relative to growing siCtrl (n=5). **D.** qPCR measuring HIRA transcripts, showing the fold change in HIRA mRNA levels relative to growing siCtrl (n=5). **E.** Representative immunofluorescence staining of HIRA (red) and RPA (green) in differentiated 9E cells transfected with siCtrl or siHIRA from three independent experiments. DAPI staining of the nucleus is shown in blue. A gray dotted line outlines the nucleus. Scale bar, 5 μm. **F.** The percentage of replication foci where HIRA is localized to HPV31 replication factories marked by RPA (n = 3; scored minimum of 23 nuclei/30 replication foci). The same siCtrl sample was utilized in Figure 5. Error bars represent ± standard deviation of the mean and shapes represent independent experiments. Statistical significance was calculated using an unpaired student's test. ns, not statistically significant, ***, P ≤ 0.001, **** P ≤ 0.0001.

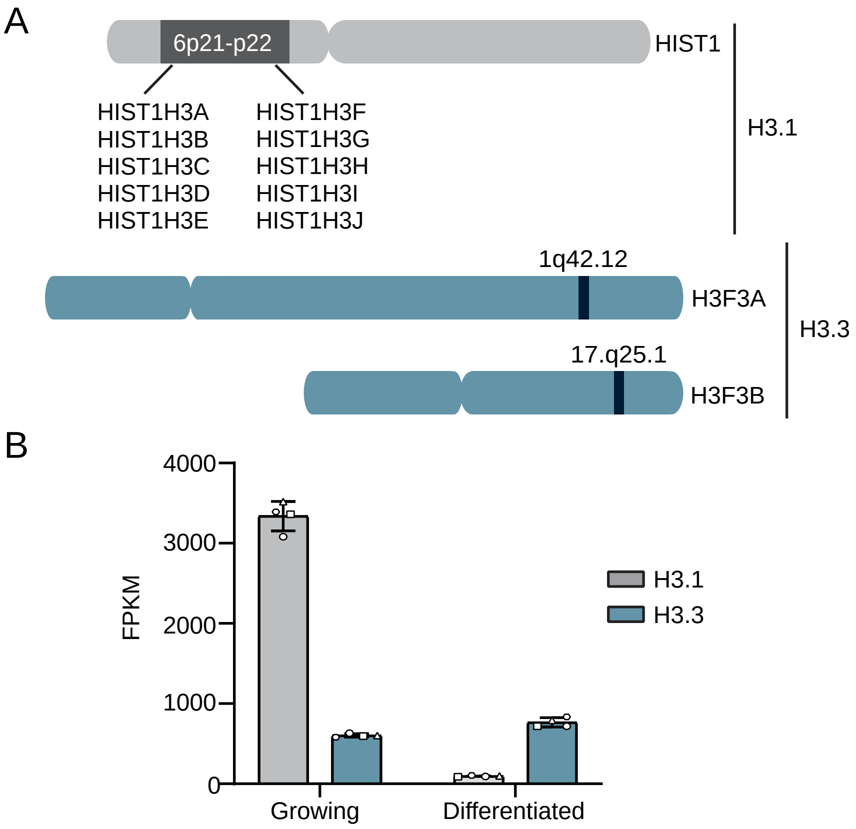

Supplementary Figure 6: Histone H3.3 is the predominant H3 histone in differentiated keratinocytes.

A. Schematic of the HIST1 gene cluster and H3F3 genes that encode histone H3.1 and H3.3 variants, respectively. The chromosome position of each gene is listed. **B.** RNA-seq analysis was performed on total RNA extracted from growing and differentiated HFK cells containing HPV16, HPV18, and HPV31 genomes described in the methods and the complete dataset will be published elsewhere. Here we show only the combined gene expression (FPKM, Fragments Per Kilobase of transcript per Million mapped reads) of genes encoding histone H3.3 and H3.1 in growing and differentiated HFKs (n=4 technical replicates). Error bars represent ± standard deviation of the mean and shapes represent independent replicate values.

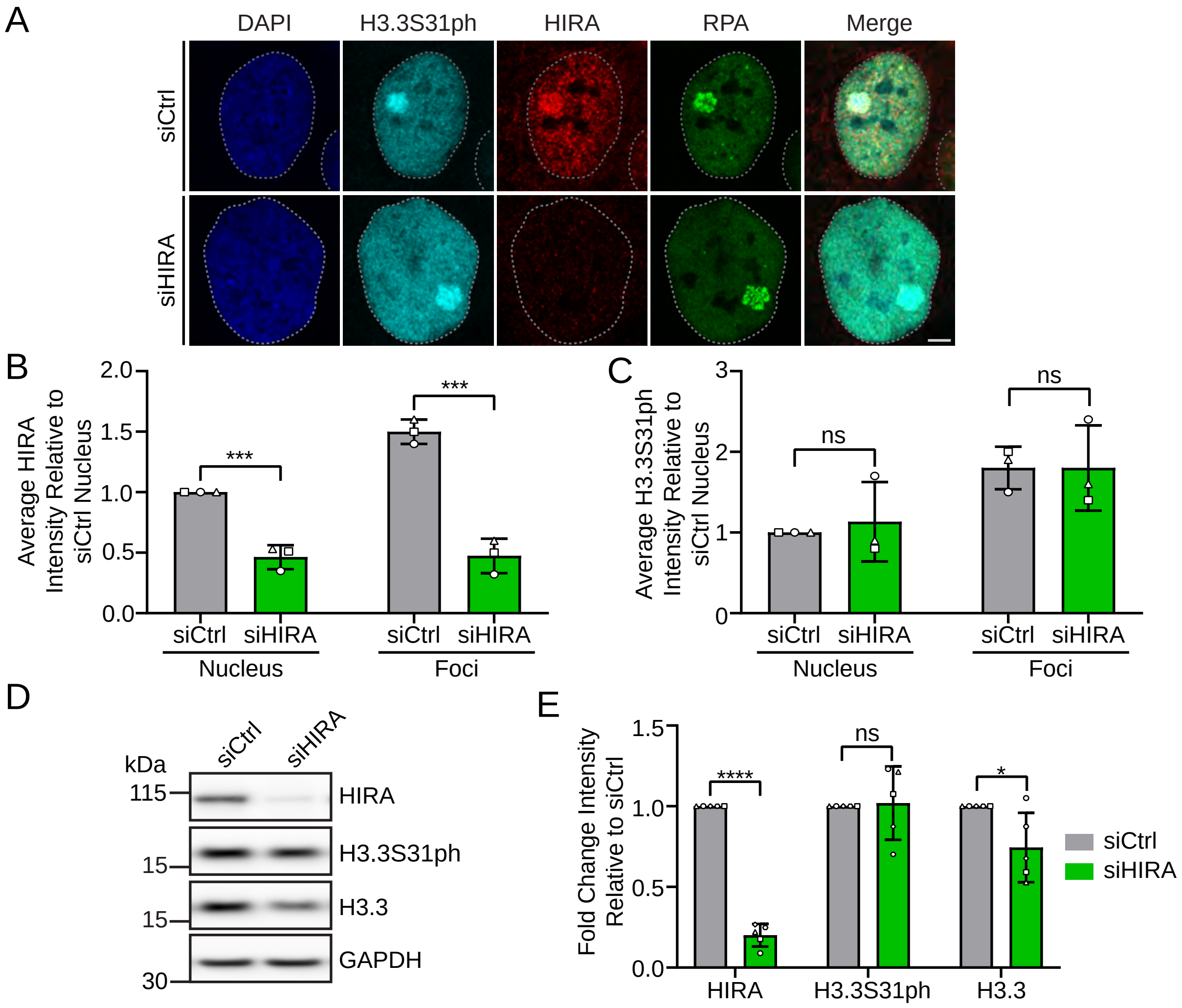

Supplementary Figure 7: Enrichment of histone variant H3.3 phosphorylated at serine 31 at HPV31 replication foci is independent of HIRA.

A. Representative immunofluorescence staining of H3.3S31ph (cyan), HIRA (red), and RPA (green) in differentiated siCtrl and siHIRA 9E cells from three independent experiments. DAPI staining of the nucleus is shown in blue. A gray dotted line outlines the nucleus. Scale bar, 5 μm. Average HIRA (**Panel B**) and H3.3S31ph (**Panel C**) intensity at RPA foci and in the nucleus relative to siCtrl nucleus was measured in ImageJ. **D.** Representative immunoblots of differentiated 9E siCtrl and siHIRA cells, visualizing the protein levels of HIRA, H3.3S31ph, H3.3, and GAPDH (n = 5). The GAPDH blot shown is representative of all blots but matched specifically to that of the H3.3 blot. **E.** Quantitation of panel D, showing the fold change in protein levels normalized to GAPDH relative to siCtrl (n= 5). Quantitation of the HIRA westerns are also represented in Supplementary Figure 4. For panel B and C quantitation, n=3; measured a minimum of 31 replication foci/18 nuclei per experiment. Error bars represent ± standard error of the mean (intensity quantitation) or standard deviation of the mean (western blot quantitation) and shapes represent independent experiments. Statistical significance was calculated an unpaired student's t-test. ns, not statistically significant, *, P ≤ 0.05, ***, P ≤ 0.001, ****, P ≤ 0.0001.

Supplementary Table 1: siRNA sequences used in this study

Target mRNA	Sequence (5' - 3')
Non-targeting Control	UGGUUUACAUGUCGACUAA
	UGGUUUACAUGUUGUGUGA
	UGGUUUACAUGUUUUCUGA
	UGGUUUACAUGUUUUCCUA
Sp100	GAAGUGAGCCUGUGAUCAA
	AGGCAUAGAUCUAAAGUAA
	UACCAGAGCCCAUGGAUUU
	GAAGGGCACUCUAUAUAAG
HIRA	CAUGGGACCCUGUUGGUAA
	GGAUAACACUGUCGUCAUC
	GCUCCGAUCCUUCCAUGUA
	GCAGGCGAUUCUGUCAAUA

Supplementary Table 2: Primers

Target	Nucleotide Position	Strand	Sequence (5' - 3')	Reference
Detection of HPV31 9E genome with qPCR	618-640	Sense	CTGACCTCCACTGTTATGAGCAA	[33]
Detection of HPV31 9E genome with qPCR	686-663	Anti-sense	CAGCTGGACTGTCTATGACATCCT	[33]
qPCR primer for (H1RNA) gene (RPPH1) on chr 14, cytoband 14q11.2.	7-26	Sense	CGGAGGGAAGCTCATCAGTG	
qPCR primer for (H1RNA) gene (RPPH1) on chr 14, cytoband 14q11.2.	95-76	Anti-sense	TGGCCCTAGTCTCAGACCTT	
qPCR primer for HPV31 E6*1	186-210, 413-416	Sense	AGATTGAATTGTGTCTACTGCAAAGGTGT	[33]
qPCR primer for HPV31 E6*1	520-498	Anti-sense	GCTATGCAACGTCCTGTCCACCT	[33]
qPCR primer for HPV31 E1^E4	857-877, 3294-3296	Sense	CTACAATGGCTGATCCAGCAGCA	[33]
qPCR primer for HPV31 E1^E4	3405-3387	Anti-sense	CGCCGCACACCTTCACTGG	[33]
qPCR primer for HPV31 L1	3562-3590, 5552-5554	Sense	TGCAACTACACCTATAATACACTTAAAAGATG	[33]
qPCR primer for HPV31 L1	5641-5619	Anti-sense	TCGTGTTACATATTCATCCGTGC	[33]
qPCR primer for Human involucrin in differentiating cells	31-53	Sense	TTACTGTGAGTCTGGTTGACAGT	
qPCR primer for Human involucrin in differentiating cells	150-128	Anti-sense	GGTATTGACTGGAGGAGGAACAG	
qPCR primer for Human filaggrin in differentiating cells	198-221	Sense	GCAAATCCTGAAGAATCCAGATGA	
qPCR primer for Human filaggrin in differentiating cells	324-303	Anti-sense	TGCTTGAGCCAACTTGAATACC	
qPCR primer for HIRA	280-299	Sense	GGGTCAACCACAATGGCAAG	
qPCR primer for HIRA	351-332	Anti-sense	CTCCAGTTGCGAACTTGGTC	
qPCR primer for Sp100, Isoform A	1594-1613	Sense	ACTTGGCCTGCAGAATGTCA	[33]
qPCR primer for Sp100, Isoform A	1676-1655	Anti-sense	CAAGGTAGTGAAGGTGCTCAGA	[33]
qPCR primer for Sp100, Isoform B	2212-2236	Sense	TCTGCCAATGTCTCGTCTATTATGT	[33]
qPCR primer for Sp100, Isoform B	2291-2263	Anti-sense	TTATGATGATGGGTCAATTTAAAGACTGT	[33]
qPCR primer for Sp100, Isoform C	2455-2472	Sense	CTGCCTGAGGAGCAGTTGAA	[33]
qPCR primer for Sp100, Isoform C	2538-2519	Anti-sense	CGGTTCTGAGGCGAAAAAGC	[33]
qPCR primer for Sp100, Isoform HMG	2224-2244	Sense	GTTGACCCTTGTGAGGAGCAT	[33]
qPCR primer for Sp100, Isoform HMG	2365-2345	Anti-sense	TGTCCGCCTTTGCCATATCTT	[33]
qPCR primer for cyclophilin PPIA	503-526	Sense	AGAACTTCATCCTAAAGCATACGG	

qPCR primer for cyclophilin PPIA	532-513	Anti-sense	TGCTTGCCATCCAACCACTC	

Supplementary Table 3: Antibodies

Target Protein	Manufacturer	Catalog Number	RRID Number	Species	IF Concentration	Western Blot Concentration	Western Blot Conditions
HIRA (WC119)*	Millipore	04-1488	AB_1977097	Mouse Monoclonal	1:100	1:500	5% Milk in TBST; 4C overnight
PML (H-238)	Santa Cruz	sc-5621	AB_2166848	Rabbit Polyclonal	1:100		
Sp100	Sigma	HPA016707	AB_1857398	Rabbit Polyclonal	1:500	1:2,000	5% Milk in TBST; RT 1 hour
UBN1	Sigma	HPA061029	AB_2684420	Rabbit Polyclonal	1:100		
ASF1a	Cell Signaling	2990s	AB_2289918	Rabbit Monoclonal	1:100		
RNA Pol II S2	Abcam	ab-5095	AB_304749	Rabbit Polyclonal	1:100		
Rad51	Abcam	ab133534	AB_2722613	Rabbit Monoclonal	1:100		
H3.3S31ph (EPR1873)	Abcam	ab92628-1001	AB_10563637	Rabbit Monoclonal	1:100	1:1,000	5% BSA in TBST; 4C overnight
H3.3	Abcam	ab176840	AB_2715502	Rabbit Monoclonal	1:200	1:500	5% Milk in TBST; 4C overnight
H3	Millipore Sigma	07-690	AB_417398	Rabbit Polyclonal		1:5,000	5% Milk in TBST; RT 1 hour
RPA32/RPA2 (4E4)	Cell Signaling	2208	AB_2927646	Rat Monoclonal	1:300		
Glu-Glu Epitope Tag	Novus	NB600-354	AB_10003158	Rabbit Polyclonal	1:300		
GAPDH (6C5)	Santa Cruz	sc-32233	AB_627679	Mouse Monoclonal		1:1,000	5% Milk in TBST; RT 1 hour
Alexa Fluor 488 H2AX (pS139)	BD Pharmingen	560445	AB_1645352	Mouse	1:10		
TopBP1 (R1180)	Boner et. al 2002			Rabbit	1:500		
TopBP1 (B-7)	Santa Cruz	sc-2710443	AB_10610636	Mouse Monoclonal	1:100		
ATR	Cell Signaling	2790	AB_2227860	Rabbit Polyclonal	1:100		
Phospho-Chk1 (Ser345) (133D3)	Cell Signaling	2348	AB_331212	Rabbit Monoclonal	1:200		

H3K27ac	Sigma	07-360	AB_310550	Rabbit Polyclonal	1:100		
p300 (3G230/NM-11)	Abcam	ab14984	AB_301550	Mouse Monoclonal	1:500		
Brd4 (1F11)	Cheng-Ming Chiang			Mouse Monoclonal	1:100		

* Antibody incubated at room temperature for 1 hour or at 4C overnight.
